# Effects of void nodes on epidemic spreads in networks

**DOI:** 10.1038/s41598-022-07985-9

**Published:** 2022-03-10

**Authors:** Kazuki Kuga, Jun Tanimoto

**Affiliations:** 1 Faculty of Engineering Sciences, Kyushu University, Kasuga-koen, Kasuga-shi, Fukuoka 816-8580 Japan; 2 Interdisciplinary Graduate School of Engineering Sciences, Kyushu University, Kasuga-koen, Kasuga-shi, Fukuoka 816-8580 Japan

**Keywords:** Mathematics and computing, Statistical physics, thermodynamics and nonlinear dynamics

## Abstract

We present the pair approximation models for susceptible–infected–recovered (SIR) epidemic dynamics in a sparse network based on a regular network. Two processes are considered, namely, a Markovian process with a constant recovery rate and a non-Markovian process with a fixed recovery time. We derive the implicit analytical expression for the final epidemic size and explicitly show the epidemic threshold in both Markovian and non-Markovian processes. As the connection rate decreases from the original network connection, the epidemic threshold in which epidemic phase transits from disease-free to endemic increases, and the final epidemic size decreases. Additionally, for comparison with sparse and heterogeneous networks, the pair approximation models were applied to a heterogeneous network with a degree distribution. The obtained phase diagram reveals that, upon increasing the degree of the original random regular networks and decreasing the effective connections by introducing void nodes accordingly, the final epidemic size of the sparse network is close to that of the random network with average degree of 4. Thus, introducing the void nodes in the network leads to more heterogeneous network and reduces the final epidemic size.

## Introduction

The spreading of infectious diseases, such as measles, influenza, Ebola, and SARS (severe acute respiratory syndrome) have threatened human societies, and now they have severe difficulties with COVID-19 (SARS-CoV2). Many mathematical models and methods have been developed to understand epidemic dynamics and the effect of preventing strategies, such as vaccination and social distancing^1–16^. For example, the degree-based mean-field model^17–20^ is the most popular mean-field approximation model with consideration of epidemic spreading in networks by using a single-order approximation, although it can be integrated to some heterogeneous topologies obeying degree distribution. Alternatively, pair approximation models explicitly express the epidemic process both at the node and at the link level. House and Keeling^21,22^ firstly developed a pair approximation model for a susceptible–infected–recovered (SIR) epidemic model with network clusters and discussed the basic reproduction number and final epidemic size. Bauch^23^ established a pair approximation for a susceptible–infected–susceptible (SIS) epidemic model and analyzed its basic reproduction number. Recently, Kuga et al.^24^ successfully established a theoretical framework of pair approximation for the vaccination game in which the both dynamic processes of epidemic spread and individual actions in helping prevent harmful social behaviors are quantitatively evaluated. These epidemic models in networks have resulted in a much better understanding of the role of contact heterogeneity and clustering of contacts. Besides these, there are many other well-established epidemic models. The edge-based compartmental model (EBCM)^25^ proposed a compact expression to capture SIR dynamics with arbitrary transmission and infection processes in configuration-like networks. On the other hand, the message-passing approach^26^ established a complicated system including a large number of integro-differential equations. Compared with the EBCM or message-passing model, pair approximation models permit a more intuitive understanding of epidemic dynamics.

For the simplicity of mathematical modeling, many studies have assumed that the disease spreading process in a network follows an exponential distribution, which is the Markovian process. In reality, disease spreading processes are far more complicated. For example, the recovery time of malaria obeys a delta distribution, whereas smallpox follows a gamma distribution^27,28^. An epidemic spreading process that does not obey an exponential distribution is called a non-Markovian epidemic process. The mathematical description, theoretical analysis, and numerical simulation of a non-Markovian process are much more complicated compared with a Markovian process. Van Mieghem et al.^29^ showed that the Weibullean recovery time strongly affects the threshold of an SIS epidemic model in networks. Cator et al.^30^ examined an SIS epidemic model with a non-exponential distribution in the infection and recovery periods and showed that the functional form of prevalence in the quasi steady state was the same as that in the Markovian SIS model. Kiss et al.^31^ derived an SIR pairwise model where the recovery time is a delta distribution and gave a general expression for the final epidemic size. Röst et al.^32^ additionally derived a new pair approximation model for gamma- and uniformly distributed infectious periods. Li et al.^33^ adapted a preventive rewiring effect to the non-Markovian SIR dynamics with a fixed infectious period. Wilkinson and Sharkey^34^ investigated the epidemic model that is network based and non-Markovian, containing classic Kermack-McKendrick, pairwise, message passing, and spatial models as special cases. They also explained how systems of delayed and ordinary differential equations can provide that upper and lower limits for the probability that an individual will be infected at a given time for the Poisson contact process and infected duration distribution. In addition, various time effects such as inter-event time and memory on infectious dynamics have been verified by infectious disease models^35,36^.

When void nodes are introduced in a certain network, the links attached to the void nodes are eliminated, and a new network is formed that exhibits a different topology from the original network. Similar to the site percolation theory, the topology of the new network depends on how many void nodes are introduced into the original network. Referring research field of the spatial prisoner’s dilemma, several precursors should be noted (see Refs.^37^^,^^38^) in which cooperation can be enhanced by considering a site-diluted lattice. This is because a void site saves a cooperator from being exploited by their neighboring defectors. Regarding epidemiology, the results of epidemic dynamics, i.e., epidemic threshold and final epidemic size, are expected to change upon changing the network topology. Wang et al.^39^ studied the effect of cutting links on the final epidemic size in complex networks using the discrete SIR epidemic model. They evaluated a simple case in which connections among individuals are randomly removed and a more complex case whereby each individual retains at least a few connections after the contact reduction. Valdez et al.^40–42^ proposed an adaptive SIR model in which a link-activation–deactivation strategy different from the link-rewiring approach is introduced into the discrete SIR model to demonstrate the social distancing effect. These published researches focused on the effect of network topology changed by cutting links and link-rewiring on the epidemic dynamics. On the other hand, the present study focus on the effect of a sparse network, where void nodes are introduced, on the epidemic threshold and final epidemic size. Furthermore, we establish how these values for the sparse network compare with other complex networks.

In this paper, we present the pair approximation models for Markovian and non-Markovian SIR dynamics in a sparse network. We establish a set of ordinary differential equations (ODEs) for the Markovian process and a set of delay differential equations (DDEs) for the non-Markovian process. We derive the implicit analytical expression for the final epidemic size and present the comparison of the final epidemic size obtained for the sparse network model and that obtained for the heterogeneous degree network model. The manuscript is organized as follows. “Model derivation” section presents a description of the model and assumptions for deductive analysis. “Discussion” section provides the deductive results and discussion. “Conclusions” section  summarizes the findings of this work.

## Model derivation

We consider a sparse network based on a regular random network with infinite nodes. These nodes represent the population and void nodes, and each node has *Q* links. Each node except the void node (*B*) has a state at any time *t*, which can be either a susceptible (*S*), infected (*I*), or recovered (*R*) state. The Markovian process is considered first. When a susceptible node connects with an infected node whose probability depends on the number of *S*–*I* pairs, *S* changes to *I* with the disease transmission rate *β*. Each infected node *I* changes to a recovered node *R* with the recovery rate *γ* that is equal to the inverse of the average recovery time. Each recovered node *R* becomes immune and is not reinfected. The notations [*X*](*t*), [*XY*](*t*), and [*XYZ*](*t*) are used to denote the expected fraction of nodes in state *X*, pairs in state *X*–*Y*, and triples in state *X–Y–Z*, respectively, where *X*, *Y*, *Z* ∈ {*S*, *I*, *R*, *B*}. All notations used in the model are summarized in Table 1.

1 $$ \frac{d}{dt}[S](t) = - \beta [SI](t), $$

2 $$ \frac{d}{dt}[B](t) = 0, $$

3 $$ \frac{d}{dt}[SS](t) = - 2\beta [SSI](t), $$

4 $$ \frac{d}{dt}[SR](t) = - \beta [ISR](t) + \gamma [SI](t), $$

5 $$ \frac{d}{dt}[SB](t) = - \beta [ISB](t), $$

6 $$ \frac{d}{dt}[BB](t) = 0. $$

**Table 1:** Notations used in this work and their meanings.

Notations	Meanings
[*S*](*t*)	Fraction of susceptible nodes at time *t*
[*I*](*t*)	Fraction of infected nodes at time *t*
[*R*](t)	Fraction of recovered nodes at time *t*
[*B*](t) = *x*	Fraction of void nodes at time *t*
[*SS*](*t*)	Number of *SS* link at time *t*
[*SI*](*t*)	Number of *SI* link at time *t*
[*SR*](*t*)	Number of *SR* link at time *t*
[*SB*](*t*)	Number of *SB* link at time *t*
[*BB*](*t*)	Number of *BB* link at time *t*
[*SSI*](*t*)	Number of *SSI* triples at time *t*
[*ISR*](*t*)	Number of *ISR* triples at time *t*
[*ISB*](*t*)	Number of *ISB* triples at time *t*
*Q*	Number of degree
$< Q >$	Average number of degree
β	Infection rate
γ	Recovery rate
*r*	Relative recovery rate
σ	Infection period
*R*_0_	Basic reproduction number
$R_{0}^{p}$	Pairwise reproduction number
*α*	Connection coefficient
μ	Coefficient to close the system

These equations are exact but unclosed. To close the system, the third-order quantities have to be expressed in terms of second-order state variables as follows^26,27^:

7 $$ [XSY] = \mu \frac{[XS](t)[SY](t)}{{[S](t)}}, $$

where *μ* is expressed as ${{(Q - 1)} \mathord{\left/ {\vphantom {{(Q - 1)} Q}} \right. \kern-\nulldelimiterspace} Q}$ because the fact that a susceptible individual *S* is known to have at least one *X* or *Y* neighbor does not change the expected number of *Y* or *X* individuals amongst the other *Q* – 1 neighbors.

Furthermore, the following constraints are required:

8 $$ [S](t) + [I](t) + [R](t) + [B](t) = 1, $$

9 $$ [SS](t) + [SI](t) + [SR](t) + [SB](t) = Q[S](t). $$

Additionally, the initial condition is hypothetically defined as:

10 $$ [S](0) = 1 - x, $$

11 $$ [B](0) = x, $$

12 $$ [SS](0) = Q(1 - x)\alpha , $$

13 $$ [SB](0) = Q(1 - x)(1 - \alpha ). $$

Here, *α* is the connection coefficient which indicates how the void nodes are distributed in the network. If the void nodes are distributed homogeneously in the network, then *α* = 1–*x*. However, if the void nodes are distributed so that the *B*–*B* link is zero, then [*SB*] = *Qx*, [*SS*] = *Q*(1–2*x*), and $\alpha = {{(1 - 2x)} \mathord{\left/ {\vphantom {{(1 - 2x)} {(1 - x)}}} \right. \kern-\nulldelimiterspace} {(1 - x)}}$, which is minimum value. Here, the relationship *x* < 0.5 must be satisfied. In addition, counting the number of effective connections through which the disease may be transmitted among the population, the average degree < *Q* > is calculated as *αQ*.

To solve this set of equations, [*SI*](*t*) from Eq. (1) is substituted into Eq. (3) to obtain:

14 $$ \frac{d[SS](t)}{{d[S](t)}} = 2\mu \frac{[SS](t)}{{[S](t)}}. $$

Using the initial conditions $[S](0) = 1 - x$ and $[SS](0) = Q(1 - x)\alpha$, the integration leads to:

15 $$ [SS](t) = Q(1 - x)\alpha \left( {\frac{[S](t)}{{1 - x}}} \right)^{2\mu } . $$

Substituting $[SI](t)$ from Eq. (1) into Eq. (4) yields:

16 $$ \frac{d[SR](t)}{{d[S](t)}} = \mu \frac{[SR](t)}{{[S](t)}} - r, $$

where $r = {\gamma \mathord{\left/ {\vphantom {\gamma \beta }} \right. \kern-\nulldelimiterspace} \beta }$ is the relative recovery rate, i.e., the inverse of the basic reproduction number *R*_*0*_.

Using the initial conditions $[S](0) = 1 - x$ and $[SR](0) = 0$, performing the integration leads to:

17 $$ [SR](t) = Qr(1 - x)\left\{ {\left( {\frac{[S](t)}{{1 - x}}} \right)^{\mu } - \left( {\frac{[S](t)}{{1 - x}}} \right)} \right\}. $$

Substituting $[SI](t)$ from Eq. (1) into Eq. (5) yields:

18 $$ \frac{d}{dt}[SB](t) = - \beta \mu \frac{[SB](t)[SI](t)}{{[S](t)}}. $$

Using the initial conditions $[S](0) = 1 - x$ and $[SB](0) = Q(1 - x)(1 - \alpha )$, performing the integration leads to:

19 $$ [SB](t) = Q(1 - x)(1 - \alpha )\left( {\frac{[S](t)}{{1 - x}}} \right)^{\mu } . $$

In the steady state ($t \to \infty$), there will be no infected individuals since they spontaneously become recovered individuals with no chance of plural infection in the present model. Therefore, the constraints in Eqs. (8) and (9) can be rewritten as:

20 $$ [S](\infty ) + [R](\infty ) = 1 - x, $$

21 $$ [SS](\infty ) + [SR](\infty ) + [SB](\infty ) = Q[S](\infty ). $$

Substituting $[SS](\infty )$, $[SR](\infty )$, and $[SB](\infty )$ from Eqs. (15), (17), and (19) into Eq. (21) yields:

22 $$ \alpha \left( {\frac{[S](\infty )}{{1 - x}}} \right)^{2\mu } - (r + 1)\frac{[S](\infty )}{{1 - x}} + (1 - \alpha + r)\left( {\frac{[S](\infty )}{{1 - x}}} \right)^{\mu } = 0. $$

Defining $s = \left( {\frac{[S](\infty )}{{1 - x}}} \right)^{{{1 \mathord{\left/ {\vphantom {1 Q}} \right. \kern-\nulldelimiterspace} Q}}}$ and considering the definition of *μ*, Eq. (22) can be written as the following algebraic equation:

23 $$ \alpha s^{Q - 1} - (r + 1)s + (1 - \alpha + r) = 0, $$

which is equivalent to

24 $$ (s - 1)\left\{ {(\alpha s^{Q - 2} + \cdots + \alpha s - (r + 1 - \alpha )} \right\} = 0. $$

The nontrivial solution is then given by:

25 $$ \alpha (s^{Q - 2} + \cdots + s^{2} + s) = r + 1 - \alpha . $$

For $s > 0$, the polynomial on the left-hand side of Eq. (25) is an increasing function of *s* that vanishes at $s = 0$ and attains the value $\alpha (Q - 2)$ at $s = 1$. Therefore, a real solution exists for $0 < s < 1$, and this is the only solution as long as $r < \alpha (Q - 1) - 1$. The phase transition that occurs at the critical relative recovery rate is expressed as follows:

26 $$ r_{c} = \alpha (Q - 1) - 1. $$

The final fractions are expressed as follows:

27 $$ [S](\infty ) = (1 - x)s^{Q} , $$

28 $$ [R](\infty ) = (1 - x)(1 - s^{Q} ). $$

These final fractions are expressed so that the fraction of void nodes is included in the system. In addition, the final epidemic size should be counted among the population 1–*x*. Therefore, the final epidemic size is expressed as 1–*s*^*Q*^.

Next, the pair approximation model for the non-Markovian process is derived considering a fixed infection period *σ* equal to 1/*γ*. Here, the number of infected nodes at time *t* is replenished by $\beta [SI](t)$ and is depleted by $\beta [SI](t - \sigma )$. Thus, the dynamics of infected nodes is expressed as:

29 $$ \frac{d}{dt}[I](t) = \beta [SI](t) - \beta [SI](t - \sigma ). $$

In addition, the deletion of the *S*–*I* link that was produced at a time (*t*–*σ*), i.e., ($\beta \mu \frac{[SS](t - \sigma )[SI](t - \sigma )}{{[S](t - \sigma )}}$), needs to be precisely calculated. However, during the time interval $(t - \sigma ,t)$, an *S*–*I* link could have changed to an *I*–*I* link since the *S* node of the *S*–*I* link was infected before the *I* node recovered. Hence, the fraction of the *S*–*I* links changing to *S*–*R* links owing to recovery is discounted. Defining the *S*–*I* links that are produced at time *t* as $\left\langle {SI} \right\rangle (t)$, the discount of the *S*–*I* links is expressed as the following evolution equation^31,32^:

30 $$ \frac{{d\left\langle {SI} \right\rangle (t)}}{dt} = - \beta \left( {1 + \mu \frac{[SI](t)}{{[S](t)}}} \right)\left\langle {SI} \right\rangle (t). $$

The integration over $[t - \sigma ,t]$ leads to:

31 $$ \left\langle {SI} \right\rangle (t) = \left\langle {SI} \right\rangle (t - \sigma )\exp [ - \int_{t - \sigma }^{t} {\beta \left( {1 + \mu \frac{[SI](u)}{{[S](u)}}} \right)du} ]. $$

Therefore, the dynamics of the *S*–*I* links is expressed as:

32 $$ \begin{aligned} \frac{d}{dt}[SI](t) & = - \beta [SI](t) - \beta \mu \frac{[SI](t)[SI](t)}{{[S](t)}} + \beta \mu \frac{[SS](t)[SI](t)}{{[S](t)}} \\ & \quad - \beta \mu \frac{[SS](t - \sigma )[SI](t - \sigma )}{{[S](t - \sigma )}}\exp [ - \int_{t - \sigma }^{t} {\beta \left( {1 + \mu \frac{[SI](u)}{{[S](u)}}} \right)du} ]. \\ \end{aligned} $$

The same constraints and initial conditions that were assumed for the Markovian process are also considered here.

To solve the non-Markovian dynamics, [*SS*] from Eq. (15) is substituted into Eq. (32) to obtain:

33 $$ \begin{aligned} \frac{d}{dt}[SI](t) & = - \beta \left( {1 + \mu \frac{[SI](u)}{{[S](u)}}} \right)[SI](t) + \beta \mu Q\alpha \left( {\frac{[S](t)}{{1 - x}}} \right)^{2\mu - 1} [SI](t) \\ & \quad - \beta \mu Q\alpha \left( {\frac{[S](t - \sigma )}{{1 - x}}} \right)^{2\mu - 1} [SI](t - \sigma )\exp [ - \int_{t - \sigma }^{t} {\beta \left( {1 + \mu \frac{[SI](u)}{{[S](u)}}} \right)du} ]. \\ \end{aligned} $$

The solution of Eq. (33) is expressed as:

34 $$ [SI](t) = \int_{t - \sigma }^{t} {\beta \mu Q\left( {\frac{[S](u)}{{1 - x}}} \right)^{2\mu - 1} [SI](u)\exp [ - \int_{u}^{t} {\beta + \beta \mu \frac{[SI](s)}{{[S](s)}}ds} ]du} . $$

Using Eq. (1), one obtains:

35 $$ [SI](t) = - \mu Q\left( {\frac{[S](t)}{{1 - x}}} \right)^{\mu } \int_{t - \sigma }^{t} {\frac{d[S](u)}{{du}}\left( {\frac{[S](u)}{{1 - x}}} \right)^{\mu - 1} \exp [ - \beta (t - u)]du} . $$

Inserting Eq. (35) into Eq. (1) results in:

36 $$ \frac{d}{dt}[S](t) = - \beta [SI](t) = \beta \mu Q\left( {\frac{[S](t)}{{1 - x}}} \right)^{\mu } \int_{t - \sigma }^{t} {\frac{d[S](u)}{{du}}\left( {\frac{[S](u)}{{1 - x}}} \right)^{\mu - 1} \exp [ - \beta (t - u)]du} . $$

Using the initial conditions $[S](0) = 1 - x$ and $[SS](0) = Q(1 - x)\alpha$, additional integrations and mathematical manipulations lead to:

37 $$ \alpha (1 - e^{ - \beta \sigma } )\left( {\frac{[S](\infty )}{{1 - x}}} \right)^{\mu } - \left( {\frac{[S](\infty )}{{1 - x}}} \right)^{1 - \mu } - \alpha (1 - e^{ - \beta \sigma } ) + 1 = 0. $$

Defining $s = \left( {\frac{[S](\infty )}{{1 - x}}} \right)^{{{1 \mathord{\left/ {\vphantom {1 Q}} \right. \kern-\nulldelimiterspace} Q}}}$ and considering the definition of *μ*, Eq. (17) can be written as the following algebraic equation:

38 $$ \alpha (1 - e^{ - \beta \sigma } )s^{Q - 1} - s - \alpha (1 - e^{ - \beta \sigma } ) + 1 = 0, $$

which is equivalent to

39 $$ (s - 1)\left\{ {(\alpha (1 - e^{ - \beta \sigma } )s^{Q - 2} + \cdots + \alpha (1 - e^{ - \beta \sigma } )s + \alpha (1 - e^{ - \beta \sigma } ) - 1} \right\} = 0. $$

The non-trivial solution is then given by:

40 $$ \alpha (1 - e^{ - \beta \sigma } )(s^{Q - 2} + \cdots + s^{2} + s) = 1 - \alpha (1 - e^{ - \beta \sigma } ). $$

Therefore, the phase transition that occurs at the critical set value of *βσ* which means basic reproduction number R_0_ is expressed as:

41 $$ (\beta \sigma )_{c} = - \ln \left( {1 - \frac{1}{\alpha (Q - 1)}} \right). $$

The final fractions are calculated via the same expressions used for the Markovian process, i.e., Eqs. (27) and (28).

Additionally, when defining the pairwise reproduction number $R_{0}^{p}$ as $R_{0}^{p} = \alpha (Q - 1)\frac{1}{1 + r}$ for Morkovian process and $R_{0}^{p} = \alpha (Q - 1)(1 - e^{ - \beta \sigma } )$ for non-Markovian process, the implicit relation between final epidemic size and $R_{0}^{p}$ can be written as follow from Eqs. (23) and (38):

42 $$ R_{0}^{p} (s^{Q - 1} - 1) - (Q - 1)(s - 1) = 0 $$

Thus, in terms of the relation between the final epidemic size and $R_{0}^{p}$, there are no difference between Markovian and non-Markovian processes^31^.

## Discussion

**Figure 1:**
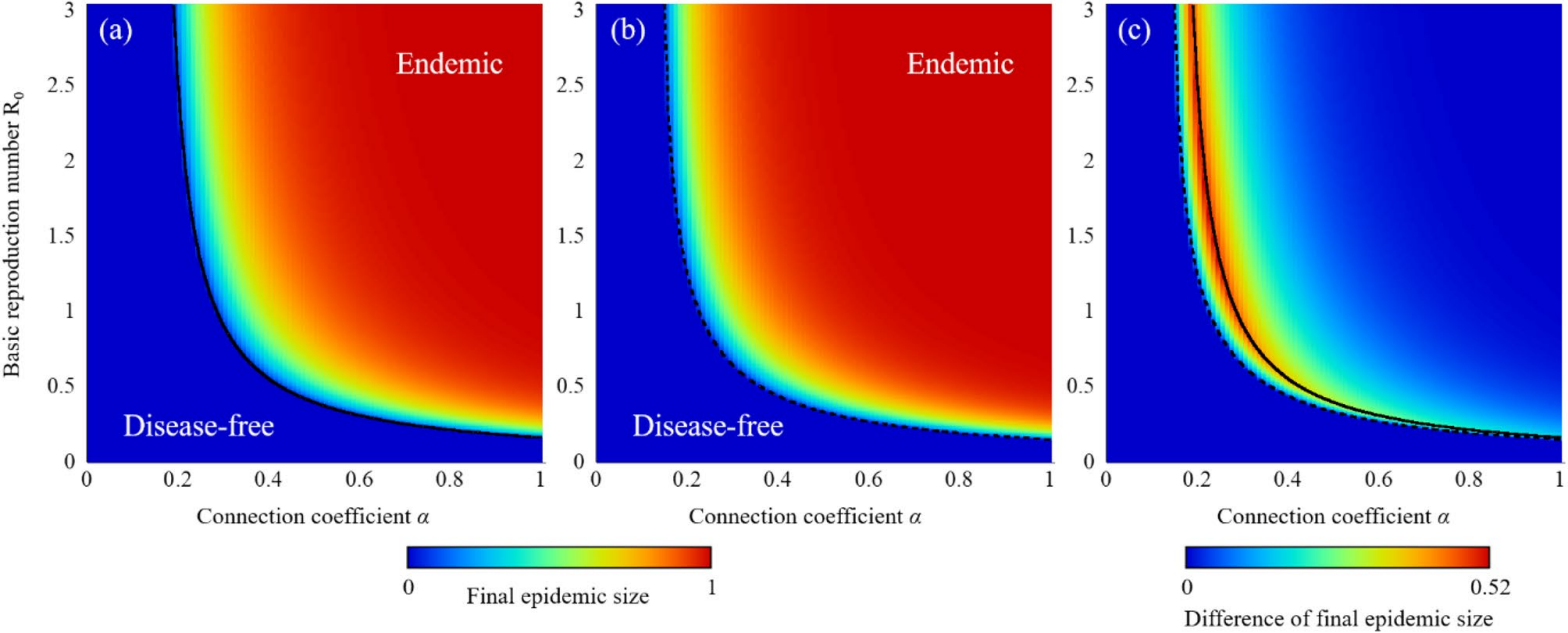
Final epidemic size corresponding to the connection coefficient *α* and infection parameters: (**a**) Markovian process with basic reproduction number R_0_ = 1/*r* = *β*/*γ* as the infection parameter; (**b**) non-Markovian process with basic reproduction number R_0_ = *βσ* as the infection parameter. Additionally, the difference of final epidemic size between Markovian and non-Markovian process is shown in (**c**). The original network is assumed to be a regular random graph with *Q* = 8. The solid line means the critical curve for Markovian process from Eq. (26) and the dot line means the critical curve for non-Markovian process from Eq. (41).

Figure 1 shows the final epidemic size corresponding to the connection coefficient *α* and infection parameters. In the Markovian process, the inverse of the relative recovery rate 1/*r* (which is equal to *β*/*γ*) was used for the infection parameter. On the other hand, *βσ* was adopted in the non-Markovian process. The both infection parameters mean the basic reproduction number *R*_0_. As the connection rate decreases from the original network connection (*α* = 1), the epidemic threshold in which epidemic phase transits from disease-free to endemic (expressed in Eqs. (26) and (41)) increases, and the final epidemic size decreases. In other words, introducing the void nodes decreases the number of effective people-to-people connections, thereby suppressing the epidemic spreading in the network. These results are consistent with previous work^39^. When comparing Markovian and non-Markovian processes, as shown in Fig. 1c, it can be noticed that the final epidemic size in the Markovian process is less than that in the non-Markovian process even if the value of the infection parameter *β*/*γ* is equal to *βσ*.

**Figure 2:**
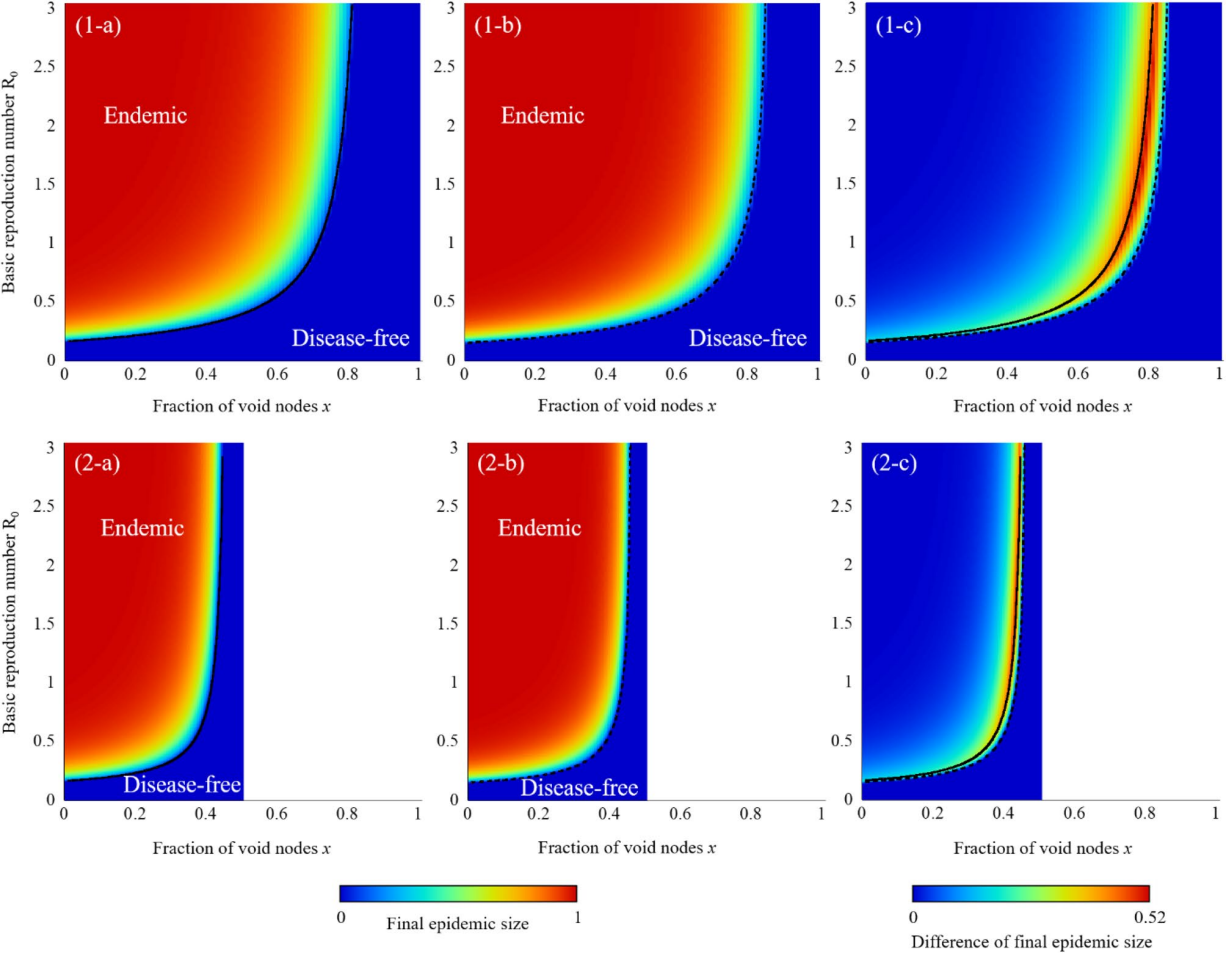
Final epidemic size according to the fraction of void node *x* and the basic reproduction number *R*_0_. (1) the case where the void node is homogeneously distributed on the network (*α* = 1 – *x*), and (2) the case where the void node is distributed so that the connection of [BB] is zero ($\alpha = \frac{1 - 2x}{{1 - x}}$). (**a**) the Markovian process, (**b**) non-Markovian process, and (**c**) difference of final epidemic size between Markovian and non-Markovian processes. The original network is assumed a regular random graph with *Q* = 8. The solid line means the critical curve for Markovian process from combination of Eq. (26) and connection coefficient *α* and the dot line means the critical curve for non-Markovian process from Eq. (41).

As shown in the mathematical expressions for the epidemic threshold (Eqs. (26) and (41)) and final epidemic size (Eqs. (23) and (38)), the fraction of void nodes *x* introduced into the original network does not explicitly affect the epidemic dynamics. However, the connection coefficient *α*, which strongly affects the final epidemic size, as shown in Fig. 1, depends on the fraction of void nodes. Therefore, the fraction of void nodes indirectly influences the final epidemic size. When the void nodes are randomly and homogeneously distributed in the network, the number of connections among individuals is inversely proportional to the fraction of void nodes, and *α* is equal to 1–*x*. As a result, Fig. 2 (1-a) and (1-b) agree well with the inverted figures in Fig. 1a,b, respectively. On the other hand, when the void nodes are efficiently distributed so that the connection of [BB] is zero and the connection of [SB] increases, the effective connection decrease obeying $\alpha = \frac{1 - 2x}{{1 - x}}$. As shown in Fig. 2 (2-a) and (2-b), the final epidemic size and epidemic threshold is significantly low compared to the case of random distribution. Moreover, the difference of final epidemic size between Markovian and non-Markovian process is shown in Fig. 2 (1-c) and (2-c). The critical curves for Markovian and non-Markovian process are derived from combination of Eqs. (26) and (41) and connection coefficient α.

**Figure 3:**
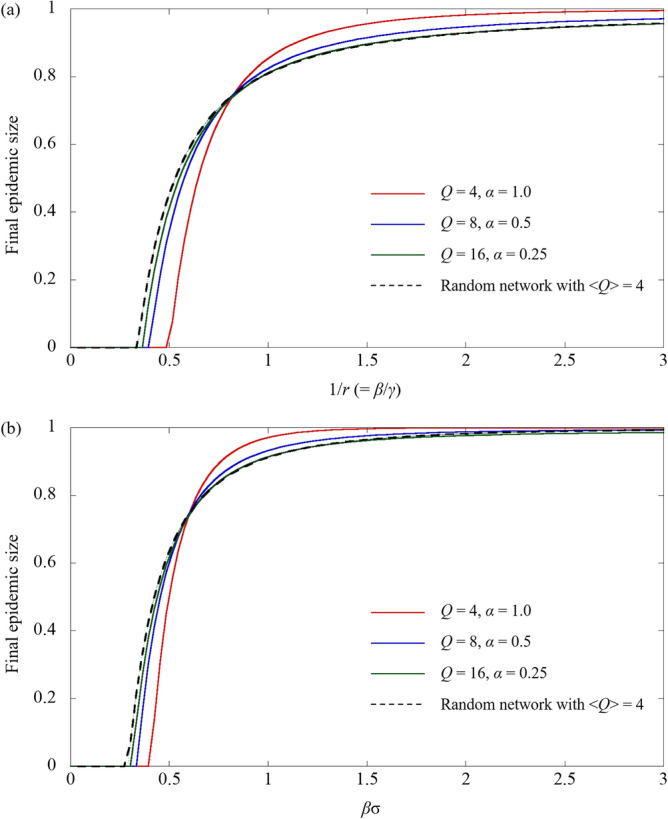
Final epidemic size as a function of (**a**) the inverse of the effective recovery rate (1/*r*) for the Markovian process and (**b**) *βσ* for the non-Markovian process.

As mentioned earlier, the introduction of void nodes can significantly change the outcome of epidemic dynamics. Furthermore, there is a question remains as to what type of network corresponds to the epidemic dynamics in a network in which void nodes are introduced in a random regular network. Erdős–Rényi (ER) random network^43^ are often used for comparison with random regular network. However, the degree distribution of the ER random network is a Poisson distribution, which is somehow heterogeneous. Therefore, the two networks are compared to investigate the effect of the heterogeneous degree distributions. To compare these results with the final epidemic size corresponding to a heterogeneous degree network, the final epidemic size associated with an ER random network was additionally derived (as described in the Supplementary Material). Figure 3 shows the final epidemic size as a function of the inverse of the effective recovery rate, i.e., 1/*r*. The network topologies are created by introducing the void nodes into different random regular networks with *Q* = 4, 8, and 16, so that the < *Q* >  = 4. In ER random networks, the degree distribution obeys the Poisson distribution with an average value of 4. In both Markovian and non-Markovian processes, upon increasing the degree of the original random regular network and decreasing the connection coefficient *α* accordingly, the final epidemic size of the sparse network is close to that of the random network with < *Q* >  = 4. In other words, by introducing void nodes into the random regular network, heterogeneous degree distribution occurred in the network with a constant degree. As a result, the epidemic dynamics of sparse network is similar to the ER random network.

## Conclusions

The pair approximation models for Markovian and non-Markovian SIR dynamics in a sparse network with the introduction of void nodes were presented. A set of ODEs for the Markovian process and DDEs for the non-Markovian process were established. The implicit analytical expression for the final epidemic size was derived, and the final epidemic size for the sparse network model and heterogeneous degree network model were found to agree with each other.

## Supplementary Information

Supplementary Information.
