# Supplementary material for: Effects of void nodes on epidemic spreads in networks
Source: Sci Rep. 2022 Mar 10;12:3957. doi: 10.1038/s41598-022-07985-9 (PMC8913681; doi:10.1038/s41598-022-07985-9)
Supplement: Supplementary file 1 — Supplementary Information. [file 41598_2022_7985_MOESM1_ESM.docx]

**Supplementary material for**

**Effects of void nodes on epidemic spreads in networks**

Kazuki Kuga^a^, Jun Tanimoto ^a,b^

^a^ Faculty of Engineering Sciences, Kyushu University, Kasuga-koen, Kasuga-shi, Fukuoka 816-8580, Japan

^b^ Interdisciplinary Graduate School of Engineering Sciences, Kyushu University, Kasuga-koen, Kasuga-shi, Fukuoka 816-8580, Japan

1. **Markovian pair approximation SIR model for heterogeneous degree networks**

We show how the Markovian pair approximation model extends to heterogeneous networks. In this case, the variables are defined as follows:

$[S_{i}](t)$: fraction of susceptible nodes of degree *i* at time *t*,

$[I_{i}](t)$: fraction of infected nodes of degree *i* at time *t*,

$[R_{i}](t)$: fraction of recovered nodes of degree *i* at time *t*,

$[S_{i}S_{j}](t)$: number of *S*–*S* links, where *S* and *S* have degrees *i* and *j*, respectively, at time *t*,

$[S_{i}I_{j}](t)$: number of *S*–*I* links, where *S* and *I* have degrees *i* and *j*, respectively, at time *t*,

$[S_{i}R_{j}](t)$:number of *S*–*R* links, where *S* and *R* have degrees *i* and *j*, respectively, at time *t*,

where *i*, *j* ∈ {*k_min_*, *k_min_* + 1, …, *k_max_*} represent the various degrees in the networks.

The Markovian pair approximation system for heterogeneous networks is expressed through the following set of ODEs:

$\frac{d}{dt}[S_{i}](t)=-\beta\sum_{k} [S_{i}I_{k}](t)$, (1)

$\frac{d}{dt}[S_{i}S_{j}](t)=-\beta[S_{j}S_{i}I](t)-\beta[S_{i}S_{j}I](t)$, (2)

$\frac{d}{dt}[S_{i}R_{j}](t)=-\beta[R_{j}S_{i}I](t)+\gamma[S_{i}I_{j}](t)$. (3)

Here, the third-order state variables $[X_{j}S_{i}I](t)$ must be converted to second-order variables for closing the system as follows:

$[X_{j}S_{i}I](t)=\mu_{i}[S_{i}X_{j}](t)\frac{\sum_{k} [S_{i}I_{k}](t)}{[S_{i}](t)}$, where $\mu_{i}=\frac{i-1}{i}$. (4)

Furthermore, the following constraints are required:

$[S_{i}](t)+[I_{i}](t)+[R_{i}](t)=P(i)$, (5)

$\sum_{k} [S_{i}S_{k}](t)+\sum_{k} [S_{i}I_{k}](t)+\sum_{k} [S_{i}R_{k}](t)=i[S_{i}](t)$, (6)

where *P*(*i*) is the fraction of nodes of degree *i* which obeys the degree distribution.

In addition, the initial condition is hypothetically defined as:

$[S_{i}](0)=P(i)$, (7)

$[S_{i}S_{j}](0)=\frac{ijP(i)P(j)}{\left\langle K\right\rangle}$, (8)

where $\left\langle K\right\rangle=\sum_{k} kP(k)$ indicates the average degree.

To solve this set of equations, $\sum_{k} [S_{i}I_{k}](t)$ from Eq. (1) is substituted into Eq. (2) to obtain the following equation:

$\frac{1}{[S_{i}S_{j}](t)}\frac{d}{dt}[S_{i}S_{j}](t)=\mu_{i}\frac{1}{[S_{i}]}\frac{d}{dt}[S_{i}](t)+\mu_{j}\frac{1}{[S_{j}]}\frac{d}{dt}[S_{j}](t)$. (9)

Using the initial conditions $[S_{i}](0)=P(i)$ and $[S_{i}S_{j}](0)=\frac{ijP(i)P(j)}{\left\langle K\right\rangle}$, the integration leads to:

$[S_{i}S_{j}](t)=\frac{ijP(i)P(j)}{\left\langle K\right\rangle}\left(\frac{[S_{i}](t)}{P(i)}\right)^{\mu_{i}}\left(\frac{[S_{j}](t)}{P(j)}\right)^{\mu_{j}}$. (10)

Adding equations for all the *S_i_–R* links, the following equation for the variable $\sum_{k} [S_{i}R_{k}](t)$ is obtained:

$\frac{d(\sum_{k} [S_{i}R_{k}](t))}{dt}=-\beta\mu_{i}\sum_{k} [S_{i}R_{k}](t)\frac{\sum_{k} [S_{i}I_{k}](t)}{[S_{i}](t)}+\gamma\sum_{k} [S_{i}I_{k}](t)$. (11)

Substituting $\sum_{k} [S_{i}I_{k}](t)$from Eq. (1) into Eq. (11) yields:

$\frac{d(\sum_{k} [S_{i}R_{k}](t))}{d[S_{i}](t)}=\mu_{i}\frac{\sum_{k} [S_{i}R_{k}](t)}{[S_{i}](t)}-r$, (12)

where $r=\gamma/\beta$ is the relative recovery rate.

Using the initial conditions $[S_{i}](0)=P(i)$ and $\sum_{k} [S_{i}R_{k}](0)=0$, the integration leads to:

$[S_{i}R](t)=irP(i)\left\{\left(\frac{[S_{i}](t)}{P(i)}\right)^{\mu_{i}}-\left(\frac{[S_{i}](t)}{P(i)}\right)\right\}$. (13)

In the steady state ($t\to\infty$), there will be no infected individuals since they spontaneously become recovered individuals. Therefore, the constraints in Eqs. (5) and (6) can be rewritten as:

$[S_{i}](\infty)+[R_{i}](\infty)=P(i)$, (14)

$\sum_{k} [S_{i}S_{k}](\infty)+\sum_{k} [S_{i}R_{k}](\infty)=i[S_{i}](\infty)$. (15)

Substituting $\sum_{k} [S_{i}S_{k}](\infty)$and $\sum_{k} [S_{i}R_{k}](\infty)$ from Eqs. (10) and (13) into Eq. (15) yields:

$\frac{1}{\left\langle K\right\rangle}\left(\frac{[S_{i}](\infty)}{P(i)}\right)^{\mu_{i}}\sum_{k} kP(k)\left(\frac{[S_{k}](\infty)}{P(k)}\right)^{\mu_{k}}+r\left\{\left(\frac{[S_{i}](\infty)}{P(i)}\right)^{\mu_{i}}-\left(\frac{[S_{i}](\infty)}{P(i)}\right)\right\}-\left(\frac{[S_{i}](\infty)}{P(i)}\right)=0$. (16)

Defining $s=\left(\frac{[S_{i}](\infty)}{P(i)}\right)^{1/i}$ and taking into account the definition of *μ*, Eq. (16) can be written as the following algebraic equation:

$\frac{1}{\left\langle K\right\rangle}\sum_{k} kP(k)s^{k-1}-(1+r)s+r=0$, (17)

which is equivalent to

$(s-1)\left\{\frac{1}{\left\langle K\right\rangle}kP(k)s^{k-2}+\frac{1}{\left\langle K\right\rangle}\left\{kP(k)+(k-1)P(k-1)\right\}s^{k-3}+...+\frac{1}{\left\langle K\right\rangle}\left\{kP(k)+...+2P(2)\right\}-(1+r)\right\}=0$.

(18)

The nontrivial solution is then given by:

$kP(k)s^{k-2}+\left\{kP(k)+(k-1)P(k-1)\right\}s^{k-3}+...+\left\{kP(k)+...+2P(2)\right\}=\left\langle K\right\rangle(1+r)$ . (19)

Therefore, the critical relative recovery rate, which defines the epidemic threshold, is expressed as:

$r_{c}=\frac{\left\langle K^{2}\right\rangle-2\left\langle K\right\rangle}{\left\langle K\right\rangle}$. (20)

1. **Non-Markovian pair approximation SIR model for heterogeneous degree networks**

Next, the final epidemic size for the nonMarkovian pair approximation SIR model is derived for heterogeneous degree networks. This system is expressed through the following set of DDEs:

$\frac{d}{dt}[S_{i}](t)=-\beta\sum_{k} [S_{j}I_{k}](t)$, (21)

$\frac{d}{dt}[S_{i}S_{j}](t)=-\beta[S_{j}S_{i}I](t)-\beta[S_{i}S_{j}I](t)$, (22)

$\begin{matrix} \frac{d}{dt}[S_{i}I_{j}](t)=-\beta\mu_{i}\frac{[S_{i}I_{j}](t)}{[S_{i}](t)}\sum_{k} [S_{i}I_{k}](t)-\beta[S_{i}I_{j}](t)+\beta\mu_{j}\frac{[S_{i}S_{j}](t)}{[S_{j}](t)}\sum_{k} [S_{j}I_{k}](t) \\ -\beta\mu_{j}\frac{[S_{i}S_{j}](t-\sigma)}{[S_{j}](t-\sigma)}\sum_{k} [S_{j}I_{k}](t-\sigma)\exp[-\int_{t-\sigma}^{t} \left(\beta\mu_{i}\frac{\sum_{k} [S_{i}I_{k}](u)}{[S_{i}](u)}+\beta\right)du] \end{matrix}$. (23)

Inserting the result of Eq. (10) into Eq. (23), one obtains:

$\begin{matrix} \frac{d}{dt}[S_{i}I_{j}](t)=-\beta\mu_{i}\frac{[S_{i}I_{j}](t)}{[S_{i}](t)}\sum_{k} [S_{i}I_{k}](t)-\beta[S_{i}I_{j}](t)+\beta\mu_{j}\frac{ijP(i)}{\left\langle K\right\rangle}\left(\frac{[S_{i}](t)}{P(i)}\right)^{\mu_{i}}\left(\frac{[S_{j}](t)}{P(j)}\right)^{\mu_{j}-1}\sum_{k} [S_{j}I_{k}](t) \\ -\beta\mu_{j}\frac{ijP(i)}{\left\langle K\right\rangle}\left(\frac{[S_{i}](t-\sigma)}{P(i)}\right)^{\mu_{i}}\left(\frac{[S_{j}](t-\sigma)}{P(j)}\right)^{\mu_{j}-1}\sum_{k} [S_{j}I_{k}](t)\exp[-\int_{t-\sigma}^{t} \left(\beta\mu_{i}\frac{\sum_{k} [S_{i}I_{k}](u)}{[S_{i}](u)}+\beta\right)du] \end{matrix}$. (24)

The solution of Eq. (24) is expressed as:

$[S_{i}I_{j}](t)=\int_{t-\sigma}^{t} \beta\mu_{j}\frac{ijP(i)}{\left\langle K\right\rangle}\left(\frac{[S_{i}](u)}{P(i)}\right)^{\mu_{i}}\left(\frac{[S_{j}](u)}{P(j)}\right)^{\mu_{j}-1}\sum_{k} [S_{j}I_{k}](t)\exp[-\int_{u}^{t} \left(\beta\mu_{i}\frac{\sum_{k} [S_{i}I_{k}](s)}{[S_{i}](s)}+\beta\right)ds]du$. (25)

Inserting Eq. (35) into Eq. (21), one obtains:

$[S_{i}I_{j}](t)=-\int_{t-\sigma}^{t} \mu_{j}\frac{ijP(i)}{\left\langle K\right\rangle}\left(\frac{[S_{i}](u)}{P(i)}\right)^{\mu_{i}}\left(\frac{[S_{j}](u)}{P(j)}\right)^{\mu_{j}-1}\frac{d[S_{j}](u)}{du}\exp[-\int_{u}^{t} \left(\beta-\mu_{i}\frac{1}{[S_{i}](s)}\frac{d[S_{i}](s)}{ds}\right)ds]du$. (26)

The variable of the exponential function in Eq. (26) is calculated as follows:

$-\int_{u}^{t} \left(\beta-\mu_{i}\frac{1}{[S_{i}](s)}\frac{d[S_{i}](s)}{ds}\right)ds=-\beta(t-u)+\mu_{i}(\ln[S_{i}](t)-\ln[S_{i}](u))$. (27)

Inserting the result of Eq. (27) into Eq. (26), one obtains:

$[S_{i}I_{j}](t)=-\frac{iP(i)}{\left\langle K\right\rangle}\left(\frac{[S_{i}](t)}{P(i)}\right)^{\mu_{i}}\mu_{j}j\int_{t-\sigma}^{t} \left(\frac{[S_{j}](u)}{P(j)}\right)^{\mu_{j}-1}\frac{d[S_{j}](u)}{du}e^{-\beta(t-u)}du$. (28)

Applying Eq. (21), one obtains:

$\frac{d}{dt}[S_{i}](t)=\beta\frac{iP(i)}{\left\langle K\right\rangle}\left(\frac{[S_{i}](t)}{P(i)}\right)^{\mu_{i}}\sum_{j} \mu_{j}j\int_{t-\sigma}^{t} \left(\frac{[S_{j}](u)}{P(j)}\right)^{\mu_{j}-1}\frac{d[S_{j}](u)}{du}e^{-\beta(t-u)}du$. (29)

The integration of Eq. (29) over [0,∞] leads to:

$\left\{\left(\frac{[S_{i}](\infty)}{P(i)}\right)^{1-\mu_{i}}-\left(\frac{[S_{i}](0)}{P(i)}\right)^{1-\mu_{i}}\right\}=\frac{\beta}{\left\langle K\right\rangle}\int_{0}^{\infty} e^{-\beta t}\sum_{j} \mu_{j}j\int_{t-\sigma}^{t} \left(\frac{[S_{j}](u)}{P(j)}\right)^{\mu_{j}-1}\frac{d[S_{j}](u)}{du}e^{\beta u}dudt$. (30)

The double integral in Eq. (30) is calculated as follows:

$\begin{matrix} \int_{0}^{\infty} e^{-\beta t}\sum_{j} \mu_{j}j\int_{t-\sigma}^{t} \left(\frac{[S_{j}](u)}{P(j)}\right)^{\mu_{j}-1}\frac{d[S_{j}](u)}{du}e^{\beta u}dudt=\sum_{j} \mu_{j}j\int_{0}^{\infty} \left(\frac{[S_{j}](u)}{P(j)}\right)^{\mu_{j}-1}\frac{d[S_{j}](u)}{du}e^{\beta u}\int_{u}^{u+\sigma} e^{-\beta t}dudt \\ =-\frac{1}{\beta}\sum_{j} \mu_{j}j\int_{0}^{\infty} \left(\frac{[S_{j}](u)}{P(j)}\right)^{\mu_{j}-1}\frac{d[S_{j}](u)}{du}e^{\beta u}(e^{-\beta(u+\sigma)}+e^{-\beta u})du \\ =\frac{1}{\beta}(1-e^{-\beta\sigma})\sum_{j} \mu_{j}j\int_{0}^{\infty} \left(\frac{[S_{j}](u)}{P(j)}\right)^{\mu_{j}-1}\frac{d[S_{j}](u)}{du}du \\ =\frac{1}{\beta}(1-e^{-\beta\sigma})\sum_{j} jP(j)\left\{\left(\frac{[S_{j}](\infty)}{P(j)}\right)^{\mu_{j}}-\left(\frac{[S_{j}](\infty)}{P(j)}\right)^{\mu_{j}}\right\} \end{matrix}$.

Inserting this integral into Eq. (30), one obtains:

$\left(\frac{[S_{i}](\infty)}{P(i)}\right)^{1-\mu_{i}}-\left(\frac{[S_{i}](0)}{P(i)}\right)^{1-\mu_{i}}=\frac{1}{\left\langle K\right\rangle}(1-e^{-\beta\sigma})\sum_{j=1}^{M} jP(j)\left\{\left(\frac{[S_{j}](\infty)}{P(j)}\right)^{\mu_{j}}-\left(\frac{[S_{j}](0)}{P(j)}\right)^{\mu_{j}}\right\}$. (31)

Using the initial condition $[S_{i}](0)=P(i)$, one obtains:

$\left(\frac{[S_{i}](\infty)}{P(i)}\right)^{1-\mu_{i}}-1=\frac{1}{\left\langle K\right\rangle}(1-e^{-\beta\sigma})\sum_{j=1}^{M} jP(j)\left\{\left(\frac{[S_{j}](\infty)}{P(j)}\right)^{\mu_{j}}-1\right\}$. (32)

Defining $s=\left(\frac{[S_{i}](\infty)}{P(i)}\right)^{1/i}$ and considering the definition of *μ*, Eq. (32) can be written as the following algebraic equation:

$\frac{1}{\left\langle K\right\rangle}(1-e^{-\beta\sigma})\sum_{j=1}^{M} jP(j)s^{j-1}-s+1-\frac{1}{\left\langle K\right\rangle}(1-e^{-\beta\sigma})=0$, (33)

which is equivalent to

$(s-1)\left\{\frac{1-e^{-\beta\sigma}}{\left\langle K\right\rangle}kP(k)s^{k-2}+\frac{1-e^{-\beta\sigma}}{\left\langle K\right\rangle}\left\{kP(k)+(k-1)P(k-1)\right\}s^{k-3}+...+\frac{1-e^{-\beta\sigma}}{\left\langle K\right\rangle}\left\{kP(k)+...+2P(2)\right\}-1\right\}=0$.

(34)

The nontrivial solution is then given by:

$(1-e^{-\beta\sigma})kP(k)s^{k-2}+(1-e^{-\beta\sigma})\left\{kP(k)+(k-1)P(k-1)\right\}s^{k-3}+...+(1-e^{-\beta\sigma})\left\{kP(k)+...+2P(2)\right\}=\left\langle K\right\rangle$. (35)

Therefore, the critical set of infection parameters *βσ*, which defines the epidemic threshold, is expressed as:

$(\beta\sigma)_{c}=-\ln\left(1-\frac{\left\langle K\right\rangle}{\left\langle K^{2}\right\rangle-\left\langle K\right\rangle}\right)$ . (36)

1. **Examples**

Here, the most typical degree of spatial structures is considered, i.e., the Poisson degree distribution which is associated with the Erdős–Rényi random graph (ER-RG). In Poisson’s degree distribution $P(k)=\exp(-\langle k\rangle)\langle k\rangle^{k}/k!$, most modes have a connectivity *k* close to the mean value $\langle K\rangle=\sum_{k} kP(k)$. Figure S1 shows the final epidemic size as a function of to the infection parameter (1/*r* for the Markovian process and *βσ* for the non-Markovian process) for different average degrees.


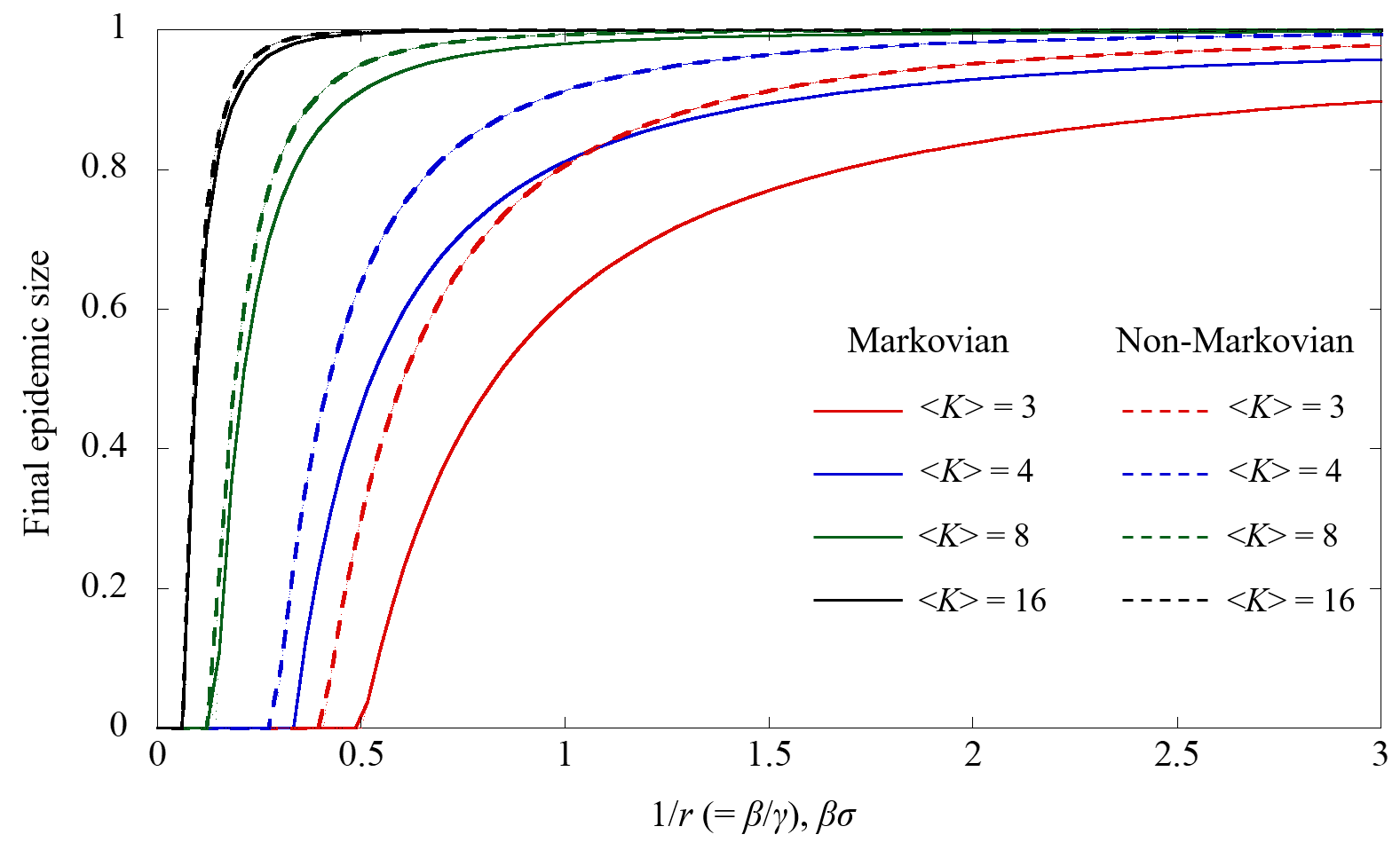


**Figure S1.** Final epidemic size as a function of the infection parameter (1/*r* for the Markovian process and *βσ* for the nonMarkovian process) for different average degrees, namely <*K*> = 3, 4, 8, and 16. The solid and dashed lines represent the Markovian and nonMarkovian processes, respectively.
